# Measuring and Visualizing Solar UV for a Wide Range of Atmospheric Conditions on Hawai’i Island

**DOI:** 10.3390/ijerph16060997

**Published:** 2019-03-19

**Authors:** Forrest M. Mims, Andrew J. S. McGonigle, Thomas C. Wilkes, Alfio V. Parisi, William B. Grant, Joseph M. Cook, Tom D. Pering

**Affiliations:** 1 Geronimo Creek Observatory, 433 Twin Oak Road, Seguin, TX 78155, USA; 2 Department of Geography, University of Sheffield, Sheffield S10 2TN, UK; a.mcgonigle@sheffield.ac.uk (A.J.S.M.); tcwilkes1@sheffield.ac.uk (T.C.W.); joe.cook@sheffield.ac.uk (J.M.C.); t.pering@sheffield.ac.uk (T.D.P.); 3 School of Geosciences, The University of Sydney, Sydney, NSW 2006, Australia; 4 Faculty of Health, Engineering and Sciences, University of Southern Queensland, Toowoomba, QLD 4350, Australia; Alfio.Parisi@usq.edu.au; 5 Sunlight, Nutrition, and Health Research Center, P.O. Box 641603, San Francisco, CA 94164-1603, USA; wbgrant@infionline.net

**Keywords:** Hawai’i, Mauna Loa Observatory, Kilauea, ultraviolet, UV Index, ozone, sulfur dioxide, smoke

## Abstract

Hawai’i Island often receives extreme (UV Index ≥ 11) solar ultraviolet radiation (UVR). While the UV Index (UVI) has been measured since 1997 at Hawai’i’s high-altitude Mauna Loa Observatory (MLO), measurements where people live and recreate are rare. We measured UVI on the face of a rotating mannequin head with UVR sensors at its eyes, ears and cheeks while simultaneously measuring the UVI with a zenith-facing sensor at MLO and seven sites at or near sea level from 19 July to 14 August 2018. The mannequin sensors received higher UVR at midmorning and midafternoon than at noon. For example, at sea level the peak UVI at the left cheek was 5.2 at midmorning and 2.9 at noon, while the horizontal UVI at noon was 12.7. Our measurements were supplemented with wide-angle (190° and 360°) sky photographs and UV images of the mannequin head. Because the UVI applies to horizontal surfaces, people in tropical and temperate latitudes should be informed that their face may be more vulnerable to UVR at midmorning and midafternoon than at noon. Finally, our instruments provided opportunities to measure unexpected UVR-altering events, including rare biomass smoke over MLO and spectroscopic measurements of substantial UVR-absorbing sulfur dioxide in the eruption plume of the Kilauea volcano.

## 1. Introduction

Solar ultraviolet radiation (UVR) has been measured by a suite of well-maintained instruments at Hawai’i Island’s high-altitude (3400 m) Mauna Loa Observatory (MLO) for over 20 years [1]. While people in Hawai’i are potentially exposed to extreme levels of UVR, we have found only one formal report giving UVR measurements where people live, work and play near sea level [2]. This report describes a 13-month field study on Hawai’i Island between December 1994 and January 1996, during which UVR was measured from near sea level at Hilo and along traverses to and from the summit of Mauna Kea (4205 m) and MLO (3400 m) on the north slope of Mauna Loa. This study used Robertson–Berger UVR radiometers (Model 501 A) [3] that measure the band of UVR wavelengths (280–400 nm) responsible for the reddening of human skin (erythema) and skin cancer. A key finding was that under a clear sky the full-sky UVR from sea level to 4205 m increased by 25 % for a solar zenith angle (SZA) of 10°.

**Figure 1:**
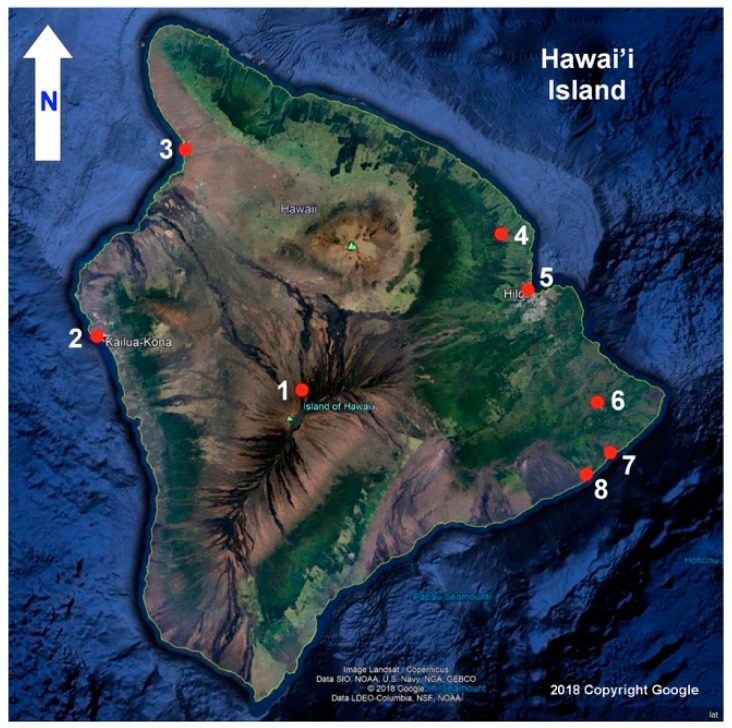
Principal ultraviolet radiation (UVR) study sites are indicated by numbered red dots on this satellite image of Hawai’i Island (unannotated image copyright by Google 2018). 1. Mauna Loa Observatory. 2. Old Kona Airport Park. 3. Spencer Beach. 4. Akaka Falls. 5. Hilo waterfront. 6. Pāhoa. 7. Seaview Estates Park. 8. Kalapana.

Our principle objective was to further this research theme by measuring the intensity of the UVI on a simulated, upright human face on Hawai’i Island at various times, places and elevations under a wide variety of sky conditions. These range from the pristine sky over MLO to skies that alter the UVI with clouds, smoke from dry season fires and vog, a haze of volcanic origin composed of gaseous SO_2_ and sulfuric acid aerosols. This objective was achieved through a field survey of erythemal UVR across Hawai’i Island from 19 July to 14 August 2018 at MLO and seven sites at or near sea level indicated in Figure 1.

Hawai’i Island was ideal for this field study because of its tropical location, the accessibility of measurement sites, the wide variety of sky conditions and the calibration opportunity provided by the suite of well-maintained UVR instruments at MLO. Our schedule specifically included the Lahaina noon on 24 July, one of two days on the Hawai’ian calendar when the sun is very near or at the zenith at solar noon when vertical posts and poles cast no shadows. This provided the opportunity to measure the UVI when the sun transited at or within a few degrees of the zenith at solar noon during its path from horizon to horizon. This very wide variety of observing conditions provides insights into UV irradiance conditions across the tropics and possibly the subtropics.

Our field study employed several very different approaches to measuring the UVI. One followed the traditional method for collecting full-sky UVR irradiance by using an instrument with a zenith-facing sensor equipped with a diffuser that provides a near-cosine response to global UVR irradiance. The second approach was to measure the UVI with sensors at the eyes, ears and cheeks of a rotating plastic mannequin head that simulated the vertical head of a standing, walking or seated person outdoors under a variety of sky conditions. Both these methods were employed simultaneously, and they were accompanied by wide-angle (190° and 360°) photographs of the sky and surrounding landscape using relatively inexpensive digital cameras as well as low cost smartphone sensor-based UV imagery to provide intuitive, color-coded visualizations of the UVR intensity across the mannequin head.

Considerable attention was given to the calibration of the UVR sensors, the measurements of which were given in terms of the widely used UVI. The rotating mannequin head apparatus and calibrated UVI sensor were relatively inexpensive (<$1500 US), easily duplicated by investigators with basic engineering skills, and sufficiently portable to be easily transported around the island. Overall, this study provides insights into the exposure to the human face of UVR under varying geographical and atmospheric conditions with relevance not only for Hawai’i Island, but also for a variety of tropical, subtropical, and high-altitude environments that are subjected to intense solar UVR.

The protocols for our study were sufficiently flexible to accommodate cloudy days and unexpected research opportunities, two of which occurred. One was the historic eruption of Kilauea Volcano [4], which was the world’s largest single source of UVR-absorbing SO_2_ during the first two weeks of our study. We used two UV cameras to reveal SO_2_ in photographs of the plume, and we measured the SO_2_ in the plume by means of a novel UV spectrometer. We also measured significant errors in total ozone measurements by a portable instrument when sunlight was passing through UVR-absorbing volcanic SO_2_. A second opportunity was the rare passage of dense forest fire smoke over MLO that permitted detailed measurements by our instruments and those at MLO of the attenuation of UVR by biomass smoke. These events are described in what follows with the intention that they will prepare others for serendipitous UVR measurement opportunities.

### 1.1. Health Effects of UVR

The UVB wavelengths (280–315 nm) stimulate synthesis of vitamin D, while both the UVB and UVA wavelengths (315–400 nm) are responsible for erythema. The intensity of these wavelengths in sunlight is influenced by scattering and absorption during their passage through the atmosphere [5]. When the sun is high in a clear sky, around half the UVR arrives from the direct sun and half is diffusely scattered by the sky. When the sun is low in the sky, diffuse UVR dominates. Natural and anthropogenic air pollution can dramatically alter this ratio and significantly reduce UVR. While UVR is absorbed by clouds, UVR scattered by cumulus clouds near the sun in an unpolluted sky can briefly increase the UVR by more than 10% [6].

More serious than UVR-initiated erythema are cutaneous malignant melanoma (CMM) and nonmelanoma skin cancers (basal cell carcinoma (BCC) and squamous cell carcinoma SCC)). The incidence of these skin cancers has latitudinal variations that depend on several factors, with the most important being UVA and UVB intensities, skin pigmentation, and sun exposure habits [7]. UVA comprises about 95% of solar UV reaching earth’s surface, with UVB comprising about 3–5% during midday in midlatitudes. UVA penetrates deeper in the skin due to the longer wavelengths, thereby being more likely to reach the bottom layer of the skin’s epidermis where melanocytes reside. Melanocytes can develop into melanomas.

Ironically, UVR also has significant health benefits. Besides suppressing pathogenic microorganisms in the outdoor environment, the UVB wavelengths stimulate the production of vitamin D in mammals and reptiles. This hormone is essential for the proper growth of bones, and insufficient UVR exposure and vitamin D in the diet can lead to the development of rickets. There is strong evidence that human skin pigmentation is a compromise between the need for protection against the adverse effects of UVR exposure and the need to produce vitamin D [8]. Moreover, geographical ecological studies have found that incidence and mortality rates for many types of cancer are lower in regions of countries with higher solar UVB doses [9]. However, since diet strongly affects risk of cancer [10], it is difficult to show that UVB does affect cancer risk when comparing rates in different countries.

Nevertheless, the damaging influence on the skin, eye, and the immune system by overexposure to UVR is widely accepted, with four out of five skin cancers being preventable [11]. This requires research as described in this paper to contribute to an improved understanding of the UVR environment to which humans are exposed.

### 1.2. The Ultraviolet Index (UVI)

In 1992, Environment Canada proposed the UV Index (UVI), a method for expressing the UVR on a horizontal surface on a scale of 0 to 10, a parameter easily understood by the public [12,13]. Because the UVI often exceeds 10 in tropical and subtropical regions, the World Health Organization [14] and World Meteorological Organization [15] removed the upper limit of 10. The UVI is now used by more than 100 countries and is widely published in the print and online media.

The UVI forecast for a day in advance is the integral of the solar UV spectral irradiance expected from ground-based UVR measurements, ground or satellite ozone measurements and cloud forecasts multiplied by the erythemal action spectrum [16,17] divided by 25 mW/m^−2^ [12,16]. This gives a nondimensional, intuitive figure of merit that is proportional to the incident UVR and much more easily understood than irradiance expressed in power per unit area.

He et al. [18] compared UVI forecasts across Canada with UVR measured by 10 Brewer spectrophotometers and found reasonable agreement, as did Cadet et al. [19] in a 10-year comparison of UVI forecasts and broadband instruments at six South African sites. Thus, while UVI forecasts are not perfect, they are considered sufficiently reliable to provide a useful guide for the public.

However, concerns have been raised about guidelines based on the UV Index. Some believe the guidelines are too strict. For example: “The important take home message for dermatologists and other clinicians is, that health campaigns promoting strict sun protection procedures to prevent skin cancer may increase the severe health risk of vitamin D-deficiency.” [20]. Others believe the guidelines aren’t sufficiently strict. For example: “Currently, health agencies recommend that no sun-protection is required when the UVI (UVI) is less than 3. We use high-quality data from spectroradiometers and model calculations to demonstrate that this simplification is seriously flawed, particularly for mid-latitude conditions.” [21]. Concerns like these led to a review of the UVI, which concluded, in part: “A number of new scientific publications have emerged since 2013 that provide indications of progress on relevant research questions, but the human evidence remains too limited to justify a modification of the WHO Global Solar UV Index and its guidance.” [22].

A well-known issue that influenced our field study and has yet to be fully addressed by the leading public health agencies is the application of the zenith-facing, horizontal standard represented by the UVI to the complex geometry of the uncovered human anatomy, the surface of which is not necessarily normal with respect to UVR arriving from the direct sun and the sky. This has been well-documented in studies that employ thin film UV sensors made from polysulfone (PS). These sensors darken over time when exposed to UVR, and this allows them to function as UVR dosimeters [23].

Attaching PS dosimeters to mannequins rather than living subjects permits UVR exposures to be measured under repeatable conditions. Downs et al. [24] and Parisi et al. [25] have applied this method by attaching PS dosimeters to various points on mannequin heads that simulate the human head and face. In the most detailed such study to date, Downs and Parisi [26] attached 1285 miniaturized PS dosimeters to a mannequin head that was exposed to sunlight over a wide range of SZAs. The dosimeter exposures were then transformed into an empirical model that provides UVR exposure maps that can be compared with the frequency and incidence of facial skin cancers.

Wang et al. [27] replaced traditional PS dosimeters with a spectrometer coupled by an optical fiber to nonhorizontal points on a rotating mannequin head. This approach has provided significant data on the exposure of a simulated human face to outdoor UVR under a variety of conditions. Of special significance is their finding that UVR on the upright mannequin face at their location (Dou Men, Zhejiang, China, 30.09° N, 120.60° E) does not peak at solar noon, when the UVI is highest on clear days. Instead, it peaks from 9:00 to 11:00 and from 13:00to 15:00 local time. Among our goals during the Hawai’i study was to replicate these findings under a variety of sun and sky conditions using simpler, less costly sensors that measure and store the UVI at 1-s intervals.

## 2. Instrumentation

Our principle instruments were a calibrated PMA1102 UVR Analog Erythema Sensor (Solar Light) to measure the horizontal UVI and a rotating mannequin head fitted with UVR sensors at its eyes, ears, cheeks and forehead. The PMA1102 sensor was inserted into a section of white PVC tube to reduce solar-induced warming. It was then placed on a platform together with its power supply (two 9-volt batteries) and a 16-bit analog data logger (Onset Hobo 4-channel analog logger) that stored voltages that represented the UVI measured by the sensor and its internal temperature. The sensor’s output voltage was converted to the UVI using the manufacturer’s algorithm, which includes a correction for the sensor’s temperature coefficient (1%/°C).

**Figure 2:**
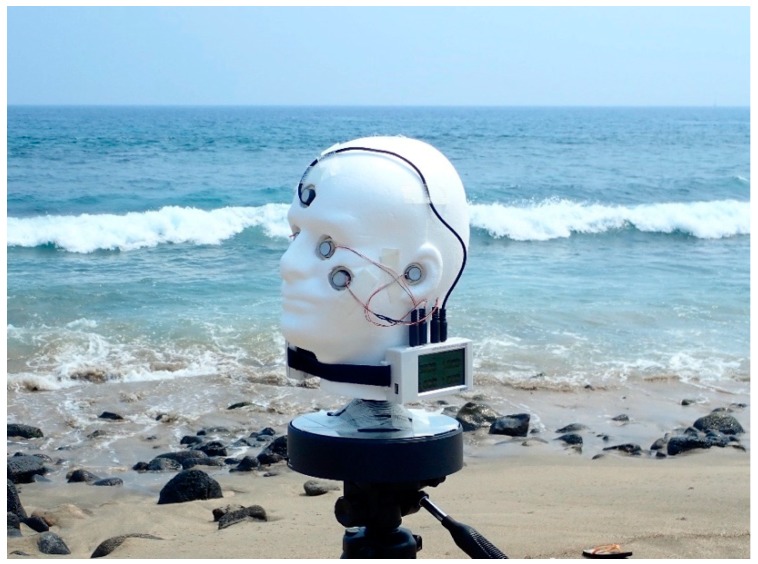
Mannequin head fitted with UVR sensors at ears, eyes, cheeks, and forehead mounted on a battery-powered turntable while collecting data at the Old Kona Airport Park beach (23 July 2018). Two 16-bit data loggers are attached to the head’s neck with VELCRO^®^. Note the nearby surf, which scattered some low level UVR toward sensors facing the ocean.

Figure 2 shows the mannequin head apparatus in operation. The heads sensors are unfiltered AlGaN photodiodes and integrated amplifier installed in a compact TO-5 enclosure (GenUV GUVB-T21GH; E-mail: uvsensor@geni-uv.com). Three-conductor phone plugs compatible with the data logger input jacks were connected to the three leads of each sensor with flexible 30 AWG wire. The sensors were then inserted into cylindrical plastic tubes fitted with 0.4-mm polytetrafluoroethylene (Teflon™) diffusers [28]. The assembled UVR sensors were inserted into the mannequin head, and their wire leads were secured to the head using fiber tape. The sensor plugs were then inserted into the input jacks of two 16-bit loggers securely strapped to the mannequin head’s neck with VELCRO^®^ Brand hook-and-loop fastening tape. The voltage from these sensors was corrected for the temperature coefficient given by the manufacturer (0.1%/°C) during postprocessing.

**Figure 3:**
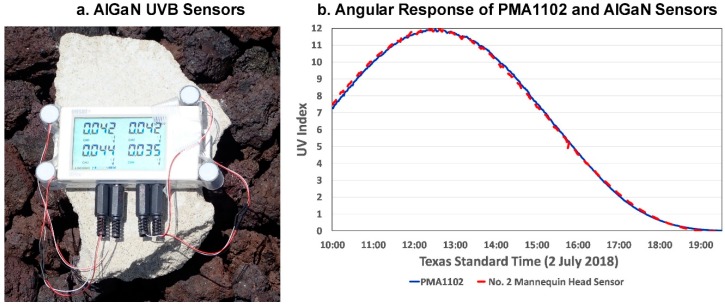
(**a**) Four AlGaN UVB sensors with Teflon™ diffusers temporarily removed from the instrumented mannequin head for a calibration check at the high-altitude (3390 3401 m MSL) Mauna Loa Observatory, and (**b**) Angular response of the PMA1102 UVR sensor and an AlGaN sensor in (**a**).

Figure 3a shows four of the UVR sensors temporarily mounted on a 16-bit data logger for a calibration check at MLO. The manufacturer’s spectral response of these sensors approximates that of the PMA1102 sensor, and the UVI measured by the PMA1102 and one of the AlGaN sensors over most of a day depicted in Figure 3b at Geronimo Creek Observatory, Texas (29.6° N 97.9° W), on 2 July 2018 is virtually identical, with the average UVI measured by the AlGaN sensor being only 0.00006 UVI higher than that measured by the PMA1102 (correlation coefficient (r^2^) = 0.999). Figure 3b affirms that the AlGaN photodiodes with Teflon™ diffusers have a cosine response that essentially matches that of the PMA1102, which the manufacturer specifies is within 5% of the ideal cosine response for angles <60°, increasing to 15% at 80°.

A comparison of the UVI measured by the PMA1102 and 4 of the AlGaN sensors at MLO (Figure 3a) the first day of our campaign (19 July) yielded high correlation coefficients (r^2^ > 0.95). These calibrations coupled with the similarity of spectral response and the very close agreement with angular response provided assurance that the UVR measured by the mannequin head sensors could be reliably compared with that measured by the laboratory calibrated PMA1102 that served as the UVI indicator during the mannequin head tests. Challenges to this assumption are given in detail in Section 4.2 of the Results and Discussion.

## 3. Methodology

The PMA1102 UVI sensor and the sensor-equipped mannequin head were deployed at seven sites during the field study (Figure 1). Four sites were at or near sea level (Hilo, Old Kona Airport Park, Spencer Beach Park, Kalapana, and Kalapana Seaview Estates Park). Sites well above sea level were Pahōa, Akaka Falls, and MLO. Table 1 lists these sites along with their coordinates, elevations, and site suitability ratings. Two locations are given for the MLO site. The instrumented mannequin head and the PMA1102 UVI apparatus were placed in an open area at the north edge of the emergency helicopter site 136 m from the MLO Solar Deck since there was no unobstructed free space on the solar deck that hosts the station’s UVR and ozone instruments.

None of the sites in Table 1 provide a totally unobstructed view of the sky. This includes MLO, which is on the north slope of Mauna Loa (3401 m) and not the summit (4169 m). Nevertheless, our 190° (2.17π sr) photographs of the sky indicate that those sites designated as Ideal or Good in Table 1 have a reasonably unobstructed view of the full sky. Clouds and weather significantly affected most of the sites. For example, while we rated the Hilo Concrete Pier off Banyan Drive as an ideal sea level site, our measurement session there was stopped by rain. Measurements at the Akaka Falls observation site ended when thick clouds arrived from the east near solar noon. Tourists from around the world were at this site, many of whom asked questions about the instrumented mannequin head and Hawai’i’s high UVI.

The night before each deployment, the three 16-bit data loggers connected to the global PMA1102 UVR sensor and the mannequin head sensors were programmed to collect data at 1-s intervals beginning at the same time the following morning. At each site, the PMA1102 platform was mounted atop a tripod and made level to within 0.2° with a bubble level. A motorized turntable powered by two 1.5-volt C cells (Master Tools 7-inch TurnTable) was mounted atop a second tripod and also made level. The mannequin head was then placed over a plastic pedestal attached atop the turntable. The turntable switch was then closed, and the mannequin commenced rotating once every 36 s or 100 times per hour. The instruments used during the field deployments and those that served as calibration standards at MLO are listed in Table 2.

During the daily measurement sessions, many photographs were made of the mannequin head and the sky. The data loggers and the cameras were downloaded into a laptop computer the evening of each measurement day, and their data was backed up to an external flash drive for safekeeping. During several days when the mannequin head was not deployed, the PMA1102 UVR sensor was mounted atop the field trip vehicle with sturdy but removable Gorilla tape.

## 4. Results and Discussion

During this 28-day study, the sun was nearly at the zenith at solar noon. While the UVR measured at and near sea level was significantly modulated by clouds and vog during many days, sky conditions allowed mannequin head deployments during some or all of 14 days. Some measurement days at MLO were made through an exceptionally clear sky in which UVR was modulated only by the ozone layer and Rayleigh scattering. During other days at MLO, clouds, vog, and a rare smoke event significantly altered the UVR. At or near sea level only a few days were free of clouds for most of a day. The results that follow reflect these very different sky conditions at the best sites where we made measurements.

### 4.1. Mannequin Head Results

**Figure 4:**
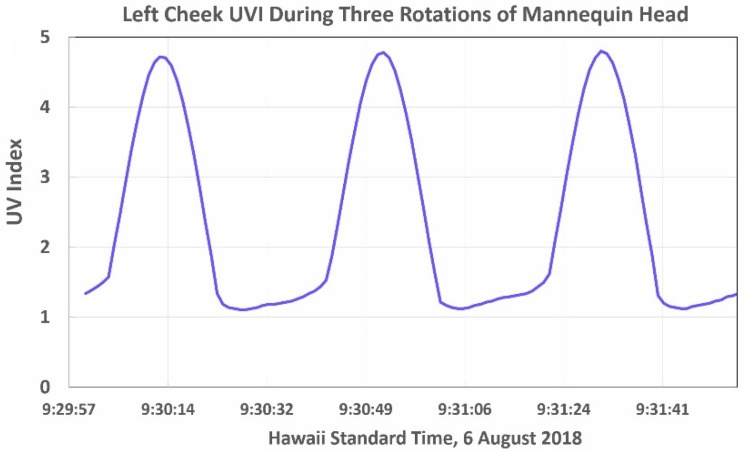
UVI measured by the mannequin head’s left cheek sensor during three revolutions at midmorning on 22 July 2018 at the Old Kona Airport park. Note the dramatic increase in the UVI as the cheek faces the sun’s direction.

When all seven head sensors were in use, the head’s two data loggers collected 420 samples each minute and 252,000 samples per 10-h day. Figure 4 shows the ozone-corrected UVI measured by the left cheek sensor during three midmorning rotations of the head. The peaks in the data occurred when the cheek was pointed toward the sun. The minima occurred when the cheek was pointed opposite the sun. While the minima have a much lower UVI than the peaks, these data provide important information about diffuse UVR scattered from the sky and the surface.

**Figure 5:**
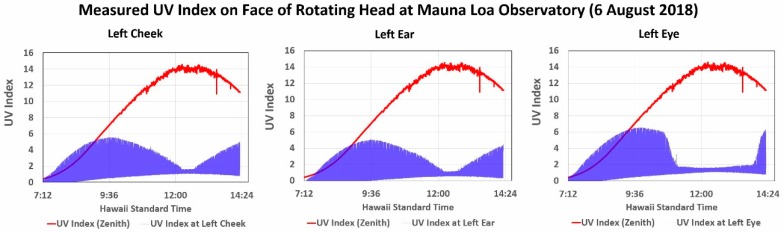
Variation in UVR measured across the face of the rotating mannequin head during a deployment at the Mauna Loa Observatory.

Figure 5 shows the variation in the ozone-corrected UVI received each second and plotted in blue for the left cheek, left ear and left eye during ~800 hundred 36-s rotations of the mannequin head at MLO on 6 August 2018. The forehead sensor was not often used, for this location is often covered by hair or shaded by a hat. The global UVR measured by the PMA1102 (red) is superimposed over the mannequin head data in Figure 5, which makes the very significant reduction in the UVI at solar noon especially obvious. At least as significant is that (1) the horizontal UVI is much higher than that measured by each of the three sensors during most of the time data was collected, and (2) the UVI measured by all three sensors is much higher at midmorning than at solar noon. As in Figure 4, the upper extreme of the ~25,000 samples plotted for each sensor in Figure 5 represents when the sensor was pointed in the direction of the sun, while the lower extreme represents when the sensor was pointed opposite the sun’s position.

Table 3 tabulates what is visually obvious in Figure 5. Note that the noon minimum UVI represents the UVR received from the sky and the lava surface, with each filling half the sensor’s FOV when the sensor was pointed opposite the direction of the sun. We address the potential significance of this scattered UVR and the cosine error of the sky in Section 4.2.2.

The UVR sensitive surfaces of the head sensors are perpendicular to the surface and pointed at the horizon, and this explains the sharp drop in the UVI at noon when the sun is almost directly overhead. While the cheek and ear sensor data form similar patterns, the eye sensor was partially shaded by the brow, and this resulted in the prolonged reduction before and after noon shown in Figure 5. Conversely, the eye sensor measured the highest UVI, and we attribute this to scattering of UVR toward the sensor by the brow. Note that the diffuse UVR scattered by the lava and sky opposite the direction of the sun was too low to be detected during the first hour or so during this measurement session.

**Figure 6:**
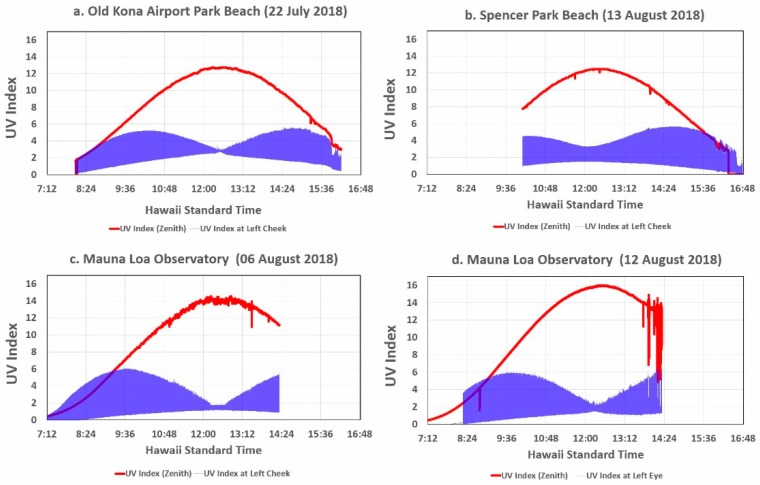
The UVI measured by the PMA1102 global UVR sensor and the mannequin head left cheek UVI sensor during the most cloud-free days at three of the observation sites.

Figure 6 shows the global UVI measured by the zenith looking PMA1102 and the mannequin head’s left cheek UVI sensor for the most cloud-free days at four of the measurement sites. The mannequin head’s left cheek was selected for comparison with the UVR measured by the PMA1102, since the cheek sensors were subject to less shading from the mannequin head’s facial features than the ear and eye sensors, which were slightly shaded by, respectively, the brows and the ear lobes.

The UVI data plotted in Figure 6 clearly show significant differences among the three sites. Table 4 summarizes the most pertinent data for the voggy Kona site in (a) and the clear MLO site in (c). The noon peak and minimum UVI is higher for Kona than MLO, which is to be expected in view of scattering of UVR from the hazy sky over Kona caused by a thick layer of vog as opposed to the very clear sky at MLO.

Note that the data plotted in Figure 6 have a much tighter waist at solar noon on 22 July and 6 August than on 12 and 13 August. This suggests that the cheek sensor was perpendicular to the surrounding surface at solar noon, while the wide waist data suggests that the sensor was looking slightly upward. This was confirmed for the 12 August session by photographs of the mannequin head, which revealed that the head was tilted upward ~10° toward the west during the morning. While the turntable was always made level before the mannequin head was placed atop it, its angle likely shifted when the mannequin head was pushed down over its holder more tightly than usual due to windy conditions.

**Figure 7:**
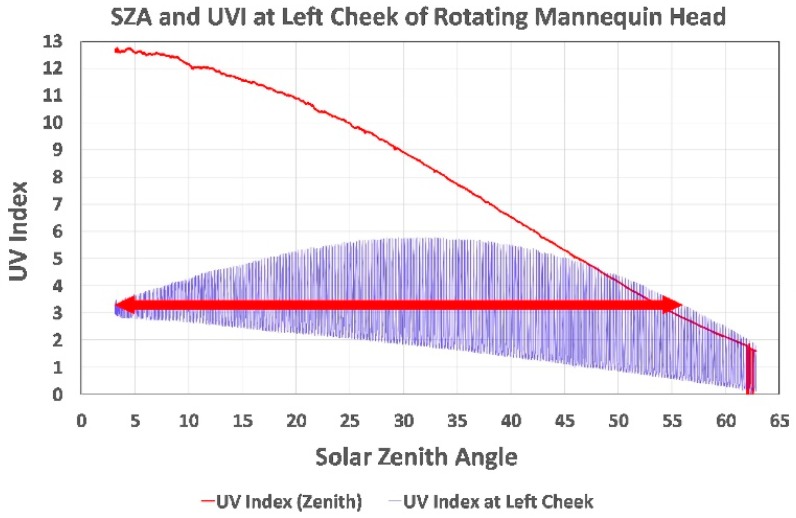
Horizontal UVI and UVI measured by the left cheek sensor plotted against the morning solar zenith angle (SZA) at the Old Kona Airport Park on 22 July 2018. The bidirectional arrow indicates that the left cheek received more UVR than at solar noon from a SZA of from 56° to 3°.

The data in Figure 4, Figure 5 and Figure 6 are plotted against local time to provide a more intuitive depiction of the UVI magnitude over the course of a day than the SZA. The data can also be plotted against the SZA as shown for the 22 July morning measurements in Figure 7. At solar noon, the PMA1102-measured UVI reached 12.8 (extremely high) while the UVI at the left cheek was only 3.3 (moderate). When the mannequin head’s left cheek was facing toward the sun, the UVR at the cheek exceeded its noon UVI (3.3) from a SZA of from 56° to 3°, reaching a peak UVI of 5.5 at a SZA of 32°, which clearly demonstrates why facial protection from UV is more important at midmorning and midafternoon than at noon for this and other tropical sites and probably temperate sites during summer.

### 4.2. UVI Measurement Challenges

We were unequipped to measure the spectral response of the UVI sensors used in this study and assumed that the responses provided by their respective manufacturers were reasonably correct. This left two challenges facing our measurements of the UVI. Here we report these challenges in detail, for they may affect those who duplicate or expand upon our study.

#### 4.2.1. Ozone Error

The first challenge was a positive error of uncertain origin in the UVI reported by the zenith-facing PMA1102 global UVR sensor. The manufacturer’s algorithm for extracting the UVI from the PMA1102 sensor’s output voltage and correcting for the temperature error produced a UVI significantly higher than that measured by the carefully maintained, temperature-regulated instruments that measure the UVI at MLO. Several of these instruments provided convenient calibration standards for checking the accuracy of the PMA1102, including two Environment Canada Brewer spectroradiometers (Nos. 009 and 119) and a Colorado State University (CSU) broadband UVR pyranometer (Yankee Environmental Systems UVB-1, Turners Falls, Massachusetts). The CSU instruments are part of the UV-B Monitoring Climatological and Research Network managed by CSU for the US Department of Agriculture [29].

For example, during the clear morning of 7 August, before the arrival of a rare smoke event at MLO, the PMA1102 indicated an average UVI 8% higher than the average UVI measured by the two Brewers and the CSU broadband radiometer. The average UVI measured by the MLO instruments agreed much more closely, with the CSU radiometer and Brewer 119 agreeing to within 0.1% and their average agreeing with Brewer 009 to within 3%.

The PMA1102 documentation established that three months before deployment to Hawai’i the sensor was calibrated by the manufacturer against an identical master sensor calibrated with a NIST-traceable standard lamp. However, the manufacturer’s calibration assumed an ozone column of 300 DU, which is higher than typical summer ozone amounts over Hawai’i. During the two hours preceding the 7 August smoke event at MLO, the average column ozone measured by Brewers 009 and 119 was, respectively, 275.2 and 276.1 DU, for an agreement of 0.3% Therefore, we added an ozone correction to the manufacturer’s algorithm in conformance with the finding by McKenzie et al. [30] that: “Ozone reductions of 1% typically cause an increase in erythemally active UV irradiance of 1.25 +/− 0.20%.” Dividing the average ozone amount (275.65 DU) by 300 DU and applying the 1.25% UVR correction factor yielded a PMA1102 UVI 3% below the average UVI measured by the three MLO instruments and 2% below the average, nearly identical UVI measured by the CSU radiometer and Brewer 119. This ozone correction was applied to all PMA1102 and mannequin head data reported herein for which the column ozone was available.

#### 4.2.2. Cosine Error

The second challenge to our study was the cosine error of the UVR sensors that we used. The cosine error is defined as: “The deviation of the angular response of a radiometer from the ideal cosine response.” [31] The cosine error of the PMA1102 sensor that provided the UVI at each site where the UVR sensor-equipped rotating mannequin head was deployed was specified by the manufacturer as within 5% for a SZA < 60°. This agrees with the sensor’s angular response error extracted from a cosine chart in the manufacturer’s data sheet, which shows the error remains <4.75% for a SZA from 20° to 70°. This range is well within the <10% for incidence angles < 60° recommended for broadband UVI radiometers by the World Meteorological Organization [31].

Finding the cosine error of the head-mounted, vertically oriented UVR photodiode sensors posed a much more difficult challenge. The angular response of the PMA1102 matched that of one of the mannequin head sensors when both sensors were mounted on a horizontal surface facing the zenith (Figure 3b). However, when the mannequin head was deployed, the PMA1102 always faced the zenith, while all the head sensors faced the horizon. Thus, the head sensors were exposed to their highest cosine error when the PMA1102 was exposed to its lowest.

Determining the cosine response error of a vertically oriented sensor at low SZAs is not trivial, especially when UVR scattered from the surface and the unpredictable, highly variable UVR scattered from the sky significantly add to the uncertainty. Apparently this challenge is not new, for a prior study in which a rotating mannequin head was used [27] does not report the cosine response of the spectrometer that served as a sensor. While flat polysulfone film UVR dosimeters would seem to have a good cosine response, the maximum SZA measured in the sole paper on this topic, to our knowledge, is 70° [32]. Evidently, a high SZA angle is subject to enhanced error caused by reflection from the surface of these sensors. Because these dosimeters generally measure integrated daily exposure, the contribution for when the sun is at a SZA between 70–90° is significantly less than when the SZA is <70° degrees.

To better understand the very high SZA cosine error of the mannequin head sensors, we looked closely at the data collected at solar noon on 22 July at the Old Kona Airport Beach, a day when the sky was made hazy by vog. Noon that day occurred at 12:30:19, when the SZA was only 3.64°. At noon the zenith-facing PMA1102 sensor measured an ozone-corrected, global UVI of 12.8, and the left cheek sensor measured an ozone-corrected UVI of 2.8. Analysis of two photographs of the left cheek established that the cheek sensor’s face was vertical with respect to the horizon formed by the ocean. Therefore, at solar noon the PMA1102 measured the total UVI from the direct sun and that scattered by the entire sky, while the vertical left cheek sensor received UVR from the sky and the surface (beach sand and ocean) but little or even none from the direct sun (see Table 4).

At 12:32 (1.7 min past solar noon) on 22 July, we measured the full and diffuse sky irradiance with a UVR (305 nm) radiometer built and used for this purpose during annual Hawaii calibrations since 1994 [33]. The full sky measurement was made by placing the instrument on the sand and making it level with the assistance of a bubble level. The diffuse sky measurement was made by shading the UVI sensor with a black, 2-cm diameter disk mounted on a 1-mm rod and held approximately 20 cm over the sensor. At the near solar noon time of these measurements, 62% of the surface UVR was diffusely received from the hazy sky and 38% from the direct sun. The sky was much clearer at MLO on 12 August at 12:23 (7.8 min before solar noon), and the same method found that only 31% of the UVR was scattered from the sky (half the sea level fraction) and 69% arrived from the direct sun. These values are conservative, for the occluder disk subtends an angle of ~5°, while the sun subtends ~0.5°. Thus, the disk blocked the most intense scattering within the solar aureole that would have added to the diffuse fraction.

Because the surface below the horizon is in the lower half of the 2.17π sr FOV of the UVR sensors on the rotating mannequin head, on 21 July (10:36) at the Old Kona Airport Park we measured the reflectance (ρ) of the dry beach sand near the mannequin head apparatus with a Model 6.5 UVI Radiometer (Solar Light). The full sky UVI was 10.1 when the instrument was pointed at the zenith, and the UVI was 0.6 when the instrument was pointed at the beach sand, which gave a ρ of 5.9% of the full sky UVI. We used a variation of this method to measure the outdoor UVR ρ of a sample of MLO lava alongside a Spectralon^®^ diffuse reflectance standard (ρ = 0.99, Labsphere, https://www.labsphere.com/), which yielded a surprisingly high ρ of 20%. These results indicate that the MLO measurements were affected less by the sky and more by surface scattering than the respective measurements made at sea level. Nevertheless, as shown in Table 4, at solar noon on 22 July the Kona site received a higher UVI (2.8) than MLO (1.6).

**Figure 8:**
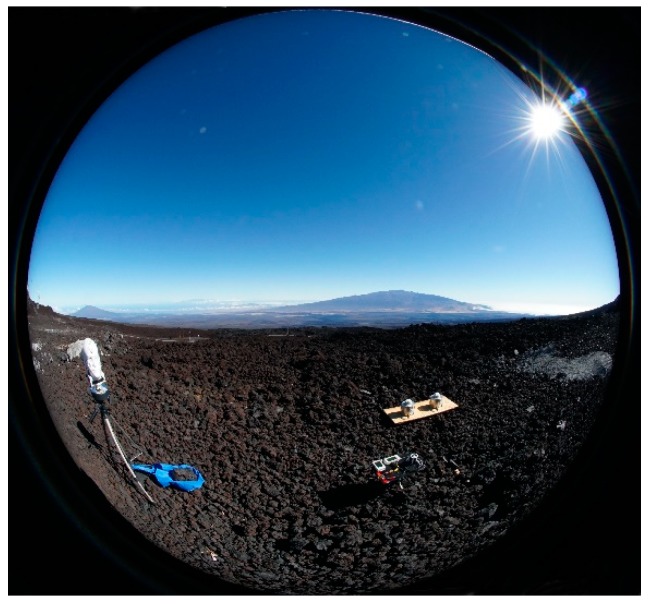
Horizontal 190° view of the sky and surface that approximates the field of view (FOV) of the mannequin head-mounted UVR sensors. This image was made at 8:52 on 11 July 2018 at MLO. The mannequin head is on the left, and the PMA1102 is to its right. Mauna Kea is in the background.

The 190° image in Figure 8 illustrates the FOV of the head-mounted sensors at MLO when pointed toward Mauna Kea. This horizontally-aligned image clearly shows how the surface and sky each form half the FOV of the vertically-aligned head sensors. We made similar images at the Old Kona Airport Park, Seaview Estates Park and Spencer Park. In retrospect, we should have made horizontal 190° images of the four ordinates at each of our sites to better document the surface conditions.

Referring to Table 5, which summarizes these measurements, note that the noon data were collected when the sun was so near the zenith that the cosine error of the sky dominated and the error for the direct sun is absent or unknown. A laboratory-tested 501 UVB radiometer (Solar Light) operated from a SZA of 0° to 85° gave a cosine error of ~0.15 at 85° [34]. This is relevant to our high SZA measurements, for the manufacturer’s cosine error for the 501 and the PMA1102 that we used are identical (<5% from a SZA of 20° to 60°). Yet, applying indoor lab measurements of cosine error is risky, for, as Hülsen and Gröbner report [35], the magnitude of the correction also depends both on the distribution of the diffuse radiation across the sky and the ratio of the direct and diffuse irradiance, neither of which is available indoors. They further observe that these uncertainties can lead to significant errors. Moreover, in our study the head sensors were continually rotating, thus exposing them to a constantly changing cosine errors from the sun and diffuse sky. While the heads of human subjects do not rotate, they often tilt up, down and sideward, thereby further complicating the cosine problem. For all these reasons, we corrected our data only for the ozone error.

### 4.3. UVR and Volcanic Sulfur Dioxide

From 1986 to 2018, continuous emissions from the Kilauea volcano caused hazy vog and respiratory distress across Hawai’i Island. Vog is formed from the aerosolization of SO_2_ that has combined with water vapor in the emission plume and the atmosphere [36]. Residual SO_2_ in vog absorbs UVR, while the aerosols in vog both absorb and scatter UVR. The latter effect significantly increases the sky’s diffuse UVR. During the first 19 days of our field study, the historic eruption of the Kilauea volcano that ended 5 August blanketed much of Hawai’i Island with considerably more vog than usual. On 22 July at the Old Kona Airport Park beach, the cloud-free sky was veiled with volcanic SO_2_ at solar noon. As explained above, byBy shading a zenith-facing, global UVI sensor with a black 2-cm diameter disk mounted on a 1-mm rod and held approximately 20 cm over the sensor, we found that 62% of the surface UVR was scattered from the hazy sky and 38% arrived from the direct sun. The sky was much cleaner at MLO on 12 August, and the same method found that only 31% of the UVR was scattered from the sky (half the sea level fraction) and 69% arrived from the direct sun. While haze can alter a UVR sensor’s cosine response, we were unable to incorporate appropriate corrections, for we did not regularly measure the diffuse/direct UVR.

**Figure 9:**
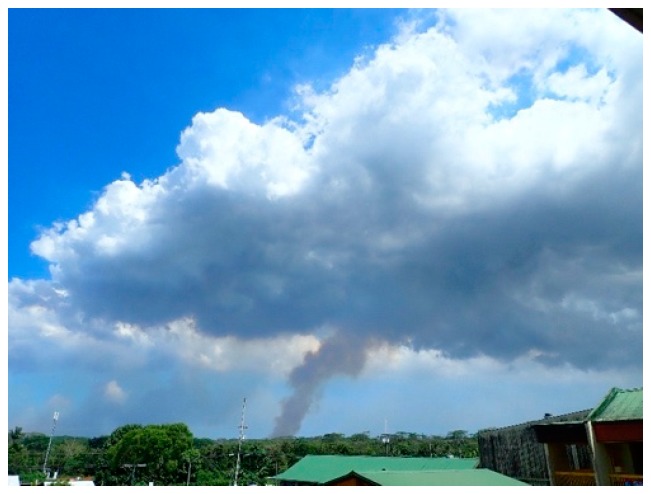
Water vapor and aerosols in the eruption plume from Fissure 8 of the Kilauea volcano created a persistent weather system that sometimes included thunderstorms. This photograph was made from a second-floor overlook at the Pāhoa High and Intermediate School on 31 July 2018.

During the main phase of this eruption, Kilauea’s fissure 8 (Figure 9) was emitting up to 50,000 tons of SO_2_ per day [37], and this provided a serendipitous opportunity to expand the measurement objectives for the Hawai’i UV study, particularly since the UVR absorption spectrum of SO_2_ emitted by coal-burning power plants and industrial activity matches the UVR spectral absorption by volcanic SO_2_. Natural and anthropogenic aerosols mixed with the SO_2_ may cause minor differences.

**Figure 10:**
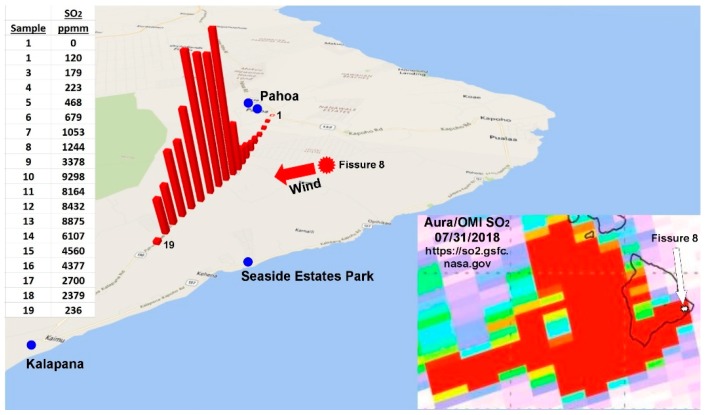
SO_2_ (ppmm) measured by a UV spectrometer during a traverse along a highway adjacent to the eruption plume from Fissure 8 of the Kilauea volcano on 31 July 2018. The inset is an Aura/Omi image of SO_2_ over and downwind from Hawai’i Island on the same day. In the inset, Kilauea’s Fissure 8 is indicated by the red dot in the large map and by a white arrow in the inset at lower right.

The Fissure 8 eruption plume created a continuous cumulus cloud infused with significant SO_2_ that we measured by pointing a PiSpec spectrometer at the zenith sky during one of two vehicular traverses along Pāhoa-Kalapana Road (Highway 130) under and adjacent to the eruption plume (Figure 10). The PiSpec is a novel UV spectrometer that has a resolution of 1 nm and a bandpass of 60 nm centered at 310 nm [38]. Specifically designed to measure volcanic SO_2_, the PiSpec is built from a 3D-printed housing, off-the-shelf optical components and a low-cost sensor primarily designed for the smartphone market. The latter is from a modified Raspberry Pi camera module, making sensor control and data acquisition relatively straightforward using the Python programming language.

Because SO_2_ efficiently absorbs UVR wavelengths also absorbed by ozone, SO_2_ from industrial sources can inflate measurements of total column ozone made by Dobson, Brewer and other spectroscopic instruments. Zerefos et al. [39] have observed that: “… SO_2_ has a number of strong UV absorption bands in which the cross-sections are three to four times as large as those for ozone.” This interference has long been known to the ozone monitoring community, and Komhyr and Evans [40] have reported that Dobson spectrophotometer measurement of total ozone in regions with high levels of anthropogenic SO_2_ can cause errors as high as +25%.

We observed examples of this while using a handheld Microtops II^®^ (Solar Light Microtops II^®^) (Solar Light, 100 East Glenside Ave., Glenside, PA 19038, USA) [41]) to make ozone measurements where the SO_2_ was especially concentrated near the Fissure 8 eruption plume. Microtops II measures direct UVR at 300, 305 and 312 nm (5 nm FWHM) in W/m^2^ when pointed directly at the sun. Microtops II infers the ozone column from ratios of direct sunlight intensity at these wavelengths, where some of the key absorption bands of SO_2_ and ozone overlap. Thus, SO_2_ can cause erroneously high ozone measurements by Microtops II. The instrument also measures total water vapor at 940 nm/1000 nm and the optical depth at 1000 nm.

**Figure 11:**
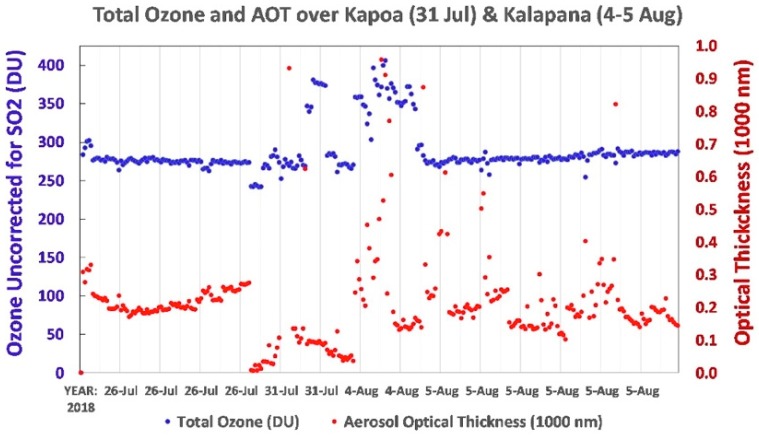
Microtops II indicated normal total ozone amounts outside the SO_2_-laden plume from Fissure 8 and highly inflated total ozone through openings in the plume.

Microtops II measurements indicated excessively high levels of total ozone over Pāhoa on 31 July and over both Kalapana and the Seaview Estates Park on 4 August. On 4 August, Microtops II measured up to 410 DU of ozone through openings to the sun in the volcanic eruption cloud over Seaview Estates Park 8 km SSW of Fissure 8. This measurement was clearly erroneous, for the average of 99 total ozone measurements from several sites well away from the eruption cloud on 24–26 July was 270 DU. Thus, it’s reasonable to conclude that the ozone error caused by SO_2_ on 4 August was 410–270 or 140 DU, an error of +52%. On 5 August at the Seaview Park site, the fraction of sky covered by the volcanic cloud was significantly reduced. This permitted 128 Microtops II total ozone measurements, the average of which was only 2% over the background ozone measured on 24–26 July. All Microtops II ozone measurements during this time are plotted in Figure 11.

The Fissure 8 eruption ceased on 5 August, and the Hawai’i Volcano Observatory described the dramatic drop in SO_2_ emissions that followed:

“When lava output from fissure 8 suddenly declined in early August, SO_2_ emission rates dropped precipitously as well. Emissions on Aug. 3 indicated tens of thousands of tons of SO_2_ coming from the fissure 8 vent, but just two days later, on Aug. 5, the emission rate was only about 200 tons per day.” [42]

### 4.4. UVR during Major Smoke Event at MLO

Brush fires caused by lightning, human activity, and military exercises are relatively common on Hawai’i Island during drought years. A rare biomass smoke event occurred over MLO from 11:45 to 12:25 on 7 August 2018 when dense smoke from the Keauhou Fire (5–15 August 2018), a major brush and forest fire on the southeast slope of Mauna Loa [43], covered most of the sky and provided a second serendipitous measurement opportunity. While this was a highly unusual event, the staff at the adjacent Mauna Loa Solar Observatory reports it has occasionally observed similar smoke events over MLO [44]. Smoke from the fire was also present over MLO on 10 August.

**Figure 12:**
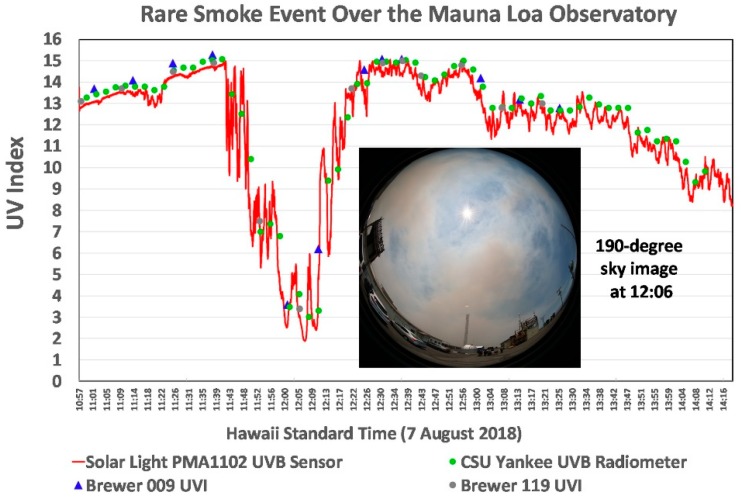
UVI measured by four instruments during a rare smoke event just before solar noon at the Mauna Loa Observatory. The Brewer data are preliminary.

UVR is strongly absorbed by smoke [45,46], and this was dramatically illustrated by the UVI measured during this event by the PMA1102 sensor and confirmed by data from a CSU broadband UVR radiometer and the pair of Canadian Brewer spectroradiometers (Nos. 009 and 119) at MLO. Figure 12 shows the UVI measured by these four sensors during the smoke event, which fortuitously arrived just before solar noon (12:30) after a very clear morning. The PMA1102 UVI data are plotted as a continuous line at one-second intervals. The CSU broadband UVI data are plotted as the average of 3-min intervals, and the preliminary UVI measured by each of the two Brewers is plotted at greater intervals. The minimum UV occurred at 12:07, when the full sky UVR was only 8.3% of the UVR before the smoke arrived. Figure 12 includes a 190° photograph of the smoky sky 1 min past the peak blockage of UVR (12:08).

**Figure 13:**
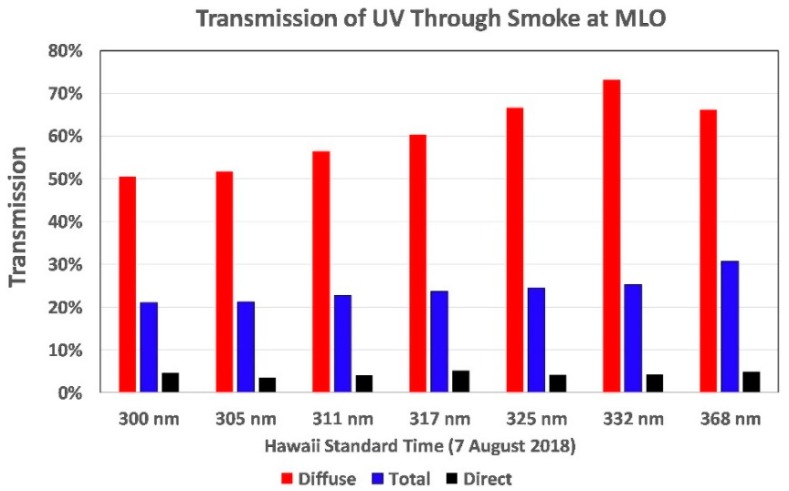
Wavelength-dependent transmission of solar UVR measured by a 7-channel rotating shadowband radiometer during a rare smoke event over the Mauna Loa Observatory.

The smoke was also measured by all 7 channels of a CSU UVR radiometer (Yankee UV-1; HI22). and a CSU 7-channel Ultraviolet Multifilter Rotating Shadowband Radiometer (Yankee UVMFR-7). The latter provides measurements at 300, 305, 311, 317, 325, 332, and 368 nm (5 nm full width, half maximum (FWHM)) of the total, direct sun, and diffuse UVR at 3-min intervals. Figure 13 shows significant attenuation of UVR at all 7 wavelengths during the smoke event. UVB wavelengths can suppress or kill exposed viruses and bacteria that might cause infectious diseases in people and animals. Thus, the persistent suppression of natural UVB by biomass smoke or clouds might play a role in the incidence of infectious respiratory diseases [46,47]. Therefore, the MLO smoke event provides relevant data for future studies of this possible connection. After the smoke drifted away, the sky became sufficiently pristine to permit Langley calibrations of 5 Microtops II sun photometers.

### 4.5. Photography

Photography provides an important and inexpensive method for recording sky conditions, yet few papers on UVR and the UVI include photographs of the sky, much less the surrounding landscape or body of water. We employed several conventional and specialized digital cameras to record the sky, landscape, rotating mannequin head, PMA1102 sensor, ocean and landscape during our measurement sessions. The principle cameras we used are listed in Table 6.

These cameras provided superb results. While their total cost was approximately $2000 US, investigators with a limited budget can collect reasonable sky images with an inexpensive mobile phone camera equipped with a clip-on, wide-angle lens available for as little as $15 US.

**Figure 14:**
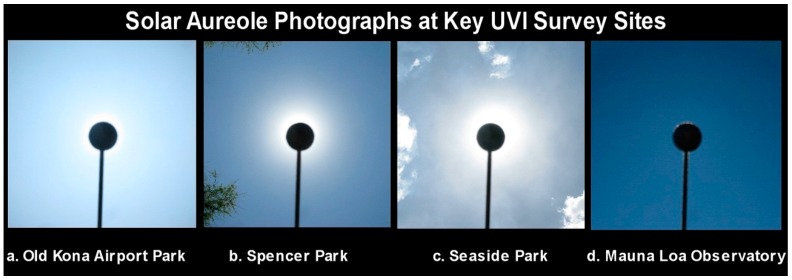
Solar aureole photographs made by blocking the sun with a black ball mounted on a digital camera [48]. The pale sky in (**a**–**c**) is caused by vog.

During deployments of the rotating mannequin head, photographs were made of the solar aureole, which provide a visually obvious view of atmospheric haze that forms a glow around the sun (Figure 14). The aureole was captured by a digital camera (Canon G9) mounted on a fixture equipped with a black ball centered in the camera’s field of view (FOV) that blocks the direct sun (0.5°) with a 4° black circle [48]. The camera settings (f4, 1/600 s, ISO-500) were identical to those used for solar aureole photographs on all days when atmospheric measurements were made at Geronimo Creek Observatory (GCO), Texas (29.6° N, 97.9° W), since 3 October 2008. These fixed settings permit quantitative comparisons of sky conditions recorded in thousands of solar aureole images from Hawai’i Island and GCO.

**Figure 15:**
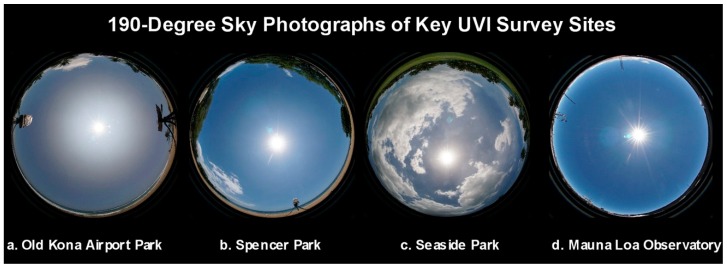
Full sky photographs at the four measurement sites in Figure 14.

The sky over each measurement site was also photographed at intervals with a digital camera (Sony α6300) equipped with a lens having a 190° (2.17π sr) FOV (Meike 6.5 mm f/2.0 Circular Fisheye Lens). These images, four of which are shown in Figure 15, show the entire sky and provide a visually obvious depiction of haze. Their outer edges show surrounding trees, soil, water, and structures.

**Figure 16:**
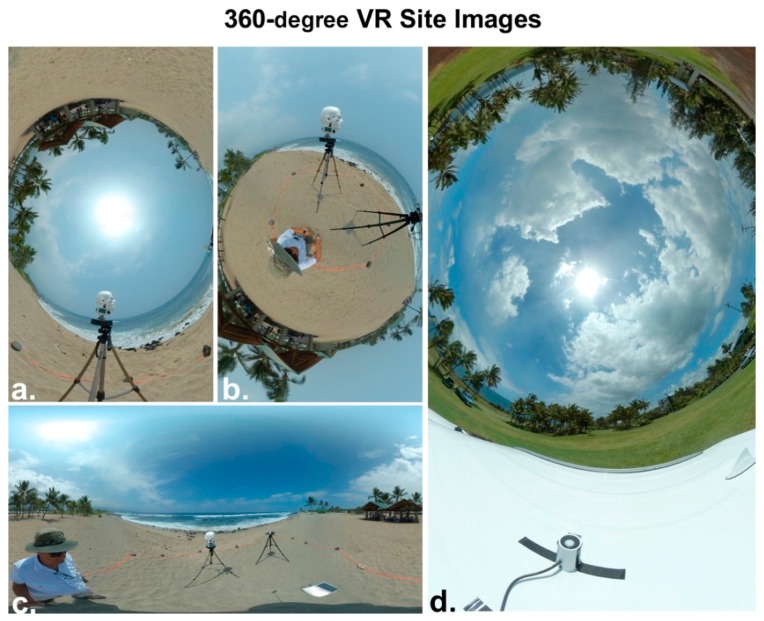
The three virtual reality (VR) images at left (**a**–**c**) were made at the Old Kona Airport Park. They show the entire sky and the trees, ground and surf surrounding the rotating mannequin head and the PMA1102 sensor. The VR image at right (**d**) shows the sky and landscape at Seaside Estates Park on 5 August 2018, the day Fissure 8 of the Kilauea volcano ceased erupting. The UVR from the sun and sky in this image was being measured by the rotating mannequin head and the PMA1102 UV sensor in the foreground, which is mounted atop the project’s rental car.

Virtual reality (VR) cameras produce omnidirectional images with a spherical, 360° (4π sr) FOV of view of the entire sky and surrounding terrain, which can be depicted in various formats (Figure 16). We employed a miniature VR camera (Xiaomi Mijia Mi Dual-Lens Sphere Action Cam) with a pair of 190° (2.17π sr) lenses, one on each side of this shirt-pocket camera. There is sufficient overlap in the FOV of the two lenses to block a rod used to support the camera, thereby providing images that suggest the camera is floating in the air.

**Figure 17:**
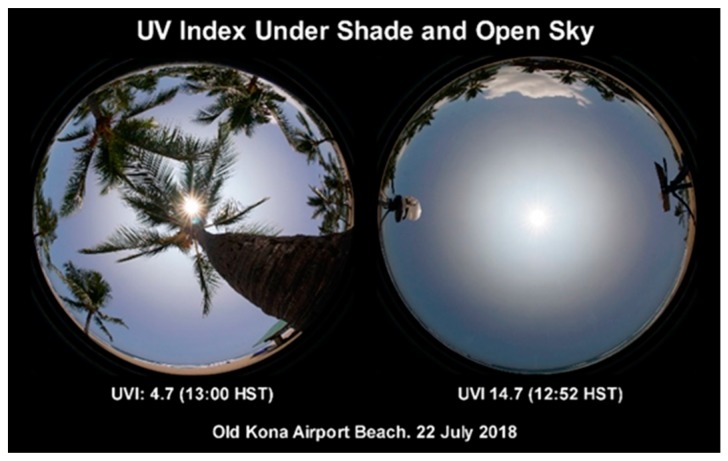
UVI in the shade of palm trees (4.7) and under an open sky (14.7) at the Old Kona Airport Park beach at near noon on 22 July 2018. The prominent solar aureole was caused by vog.

Our cameras captured images of the sky intended for a webpage for the public that describes how shade and a hazy sky affect UVI. While the role of shade in reducing the UVI is intuitive, the image of the sky through coconut palms in Figure 17 shows that significant UVR can leak through overhead branches. The public may not know that diffuse UVR scattered from a sky polluted with aerosols can exceed direct sun UVR. This is also illustrated in Figure 17, a photo of the open sky veiled by thick haze caused by volcanic vog. UVR from the diffuse sky in this image was 62% of the total UVR, while only 38% arrived from the direct sun.

**Figure 18:**
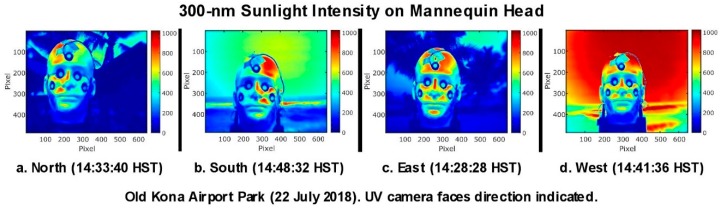
These color-coded UVR images provide a visually obvious depiction of the UVR intensity at 300 nm across the mannequin head’s face. The forehead and nose are the most consistently illuminated areas.

A novel, low-cost UV camera [49] that could be fitted with various UVR filters was used to make several hundred UV photos of the mannequin head at the Old Kona Airport Park beach. The ozone column blocks virtually all sunlight below 295 nm. Thus, the UV camera’s spectral response with a 300-nm filter (40 nm FWHM) approximated that of the UVB portion of the erythemal action spectrum. The color-coded versions of these images (TW) in Figure 18 clearly indicate significant variations in the intensity of the UVR illuminating the mannequin’s head’s face at midafternoon when the head was pointed north, east, south, and west. While we have yet to analyze these images, we include them here to illustrate their potential merit.

**Figure 19:**
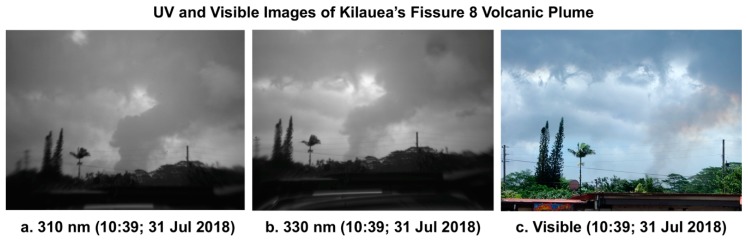
The darkened tint of UVR images (**a**,**b**) clearly reveal the presence of significant SO_2_ in the volcanic plume emerging from Kilauea’s Fissure 8. Aerosols alone are responsible for the visibility of the plume in the visible image (**c**).

Finally, the historic eruption of Kilauea provided an opportunity to employ the UV camera technique commonly applied in volcanology [50,51,52] to make UV photographs of the major emission plume from what was then the world’s single largest emitter of SO_2_, which strongly absorbs UVR. The plume from Kilauea’s Fissure 8 is clearly visible in the UVR photographs in Figure 19a,b, both of which were made at 10:39 on 31 July 2018. Photograph (a) was made through a 310-nm filter (10 nm FWHM), a wavelength strongly absorbed by SO_2_. Photograph (b) was made through a 330-nm filter (10 nm FWHM), a wavelength weakly absorbed by SO_2_. This explains why the plume appears darker and larger in the 310-nm image. Some of the dark cloud to the left (east) of the plume is caused by SO_2_ rising from the lava flowing to the ocean from Fissure 8. Figure 19c is a visible light photograph made at the same time as the two UVR images. SO_2_ does not significantly absorb visible light. Thus, the plume appears much narrower and less dense than in the UVR images.

**Figure 20:**
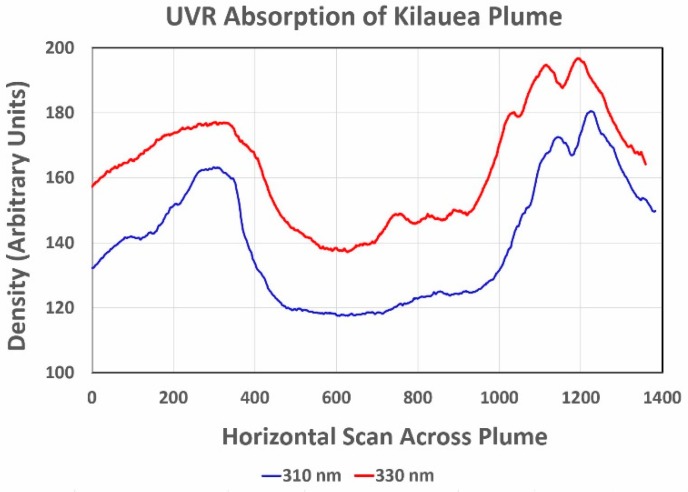
Densitometry scans across the Kilauea plume in the 310- and 330-nm images in Figure 19 show the enhanced absorption by SO_2_ at 310 nm.

Horizontal scans with ImageJ image processing software across the plume between the tops of the palm tree at left and the utility pole at right in Figure 19a,b reveal the optical density of the 310- and 330-nm images. The resulting plots in Figure 20 show the enhanced absorption by SO_2_ at 310 nm that quantifies what is visually evident in Figure 19.

## 5. Conclusions

Our findings provided new insights into UVI under various sky conditions during our brief, 28-day field campaign across Hawai’i Island. We validated previous findings regarding the intensity profile of UVR on a simulated human face [27] with an easily constructed, economical rotating mannequin head equipped with UVR sensors that can be employed under a wide variety of outdoor conditions. We demonstrated that wide-angle, visible photography of the full sky and surrounding landscape and UV photography of a mannequin head illuminated with natural sunlight can inform studies of UVR measurements. The methods and protocols reported herein can be applied by professional and academic scientists with limited research budgets.

In particular, our mannequin head findings are applicable from the tropics to the temperate latitudes, for the rotational profiles of the mannequin head measurements by Wang et al. [27] at 30.01° N (May 27, 30, 2010, minimum SZA = 8°) resemble ours first made at Geronimo Creek Observatory, Texas, at 29.61° N (6 July 2018, minimum SZA = 6°) 15 days prior to the first Hawai’i test.

We believe that the UVI provides valuable, easily understood information for the public. The UVR measurements during this study suggest that individuals who rely on the UVI to govern their UVR exposure, especially in the tropics and subtropics where extreme levels of UVR prevail, will benefit from an expanded explanation of the factors that alter the Index. For example, haze and clouds often and unpredictably reduce direct UVR while increasing diffuse UVR. These effects often altered the UVI during the present study and lend support to Blumthaler’s suggestion [53] that the public would be better served if the daily UVI forecast could be updated in real time on the World Wide Web.

We particularly agree with the observation by McKenzie et al. [54] that: “There are problems with the way UV information is presented. … the complex 3D geometry of a human might need to be taken into account.” Our mannequin head UVR sensors measured significantly diminished UVI on the cheeks, ears and eyes of the simulated face of a person standing, walking or seated outdoors at noon when the downwelling UVR is highest. Populations subject to high UVI levels should be informed that the UVI applies more to reclining people (e.g., lying on a beach) whose bodies are much more exposed to sunlight at solar noon than the faces of people who are walking, standing, or seated outdoors. This information is not provided in the UVI publications intended for the public that we have reviewed and could be easily added in a few lines of text, perhaps alongside a schematic figure of a vertical human face receiving less UVR when the sun is high in the sky than when it is somewhat lower.

While photographs of the sky are uncommon in published UVR studies, the visible light 190° and 360° VR photos included in this study illustrate the value of photography in providing scientifically useful information about the presence of clouds, haze, trees, structures, water, ocean surf, soil, and ground cover, all of which can enhance or reduce UVR. Such photographs can also better inform the public about the circumstances that influence the UVI. We are hopeful that our use of sky and landscape photography will be emulated by other UVR investigators. Furthermore, while UV cameras are highly specialized, we learned that color-coded imagery from a UV camera can provide important information about the distribution of UVR across the simulated human face provided by a mannequin head.

This study encountered two unexpected natural phenomena not included in our research plan. The first was significant SO_2_ from an historically persistent and immense volcanic plume. Because ozone and SO_2_ have similar UVR absorption spectra, SO_2_ emissions from industrial sources and thermal power plants can falsely inflate surface-based measurements of the total ozone column. Our measurements found that volcanic SO_2_ can cause a similar result. On 4 August, Microtops II measured up to 410 DU of ozone through openings to the sun in the volcanic eruption cloud when the average of 99 total ozone measurements from several sites well away from the eruption cloud on 24–26 July was 270 DU. Thus, volcanic SO_2_ inflated our ozone readings by up to 52% (Figure 11).

The second unexpected event occurred shortly before noon on 7 August when a massive plume of forest fire smoke drifted over MLO and its suite of UVR and ozone instruments. This was an especially unusual event, for MLO is an alpine site famous for its very clear skies. The average UVI measured before, during and after the smoke event by the PMA1102 UVR sensor (14.0) was remarkably close to that measured by a CSU broadband radiometer standard (14.06), Brewer 009 (14.5) and Brewer 119 (14.05). Particularly notable is that these measurements were made from a maximum SZA of 26.7° to a minimum of just 3°. These results are plotted in Figure 12, and they give confidence to the ozone-corrected calibration that we used for the PMA1102.

While our schedule was sufficiently flexible to measure and photograph these highly unusual phenomena, we neglected to make horizontal 190° photos (Figure 8) of the four ordinates at each measurement site. Those photos that we did make clearly illustrate the role, if not the magnitude, of the surface and the sky as the mannequin head rotated. We also neglected to make periodic diffuse sky UVI measurements at every site beyond one at MLO (31%), one at Kona (62%), and one at Spencer Beach (48%). Together, these missing images and data would likely have contributed toward a much better understanding of the role of the ever-changing diffuse component of the data as the head rotated. Accordingly, we propose to address these issues in a future study, while acknowledging that the sky and the natural environment are so variable that there will be no simple answers.

## Figures and Tables

**Table 1:** Principal UV Index (UVI) measuring sites for Hawai’i Island UVR study in order of their suitability.

Site	Lat°	Lon°	Elev (m)	Comments
MLO (mannequin)	19.537	−155.575	3389	Ideal. Helicopter landing zone.
MLO (Solar Deck)	19.536	−155.576	3401	Ideal. MLO UVR and ozone instruments.
Hilo Concrete Pier	19.727	−155.070	3	Ideal sea level site (trees 47 m).
Old Kona Airport	19.644	−156.011	0	Good sea level site (coconut palm 26 m).
Spencer Park Beach	20.024	−155.823	0	Good sea level site (tree 32 m).
Seaview Estates Park	19.399	−154.920	26	Good (coconut grove 40 m).
Akaka Falls	19.856	−155.152	335	Fair. Low trees and hundreds of tourists.
Kalapana	19.362	−154.967	7	Poor (2 coconut palms 15 m).
Pahōa	19.491	−154.942	218	Poor (1-story buildings and power line).

**Table 2:** Instruments used in the UVI survey and those that served as calibration standards at Mauna Loa Observatory (MLO).

**UVI Survey Instruments**	**Purpose**
PMA1102 UVR Sensor	Measurements of UVI during mannequin head sessions
AlGaN Photodiode UVR Sensors	Measurements of UVI at face of mannequin head
Microtops II Ozonometer	Total ozone and optical depth
Custom UVR radiometer	Direct sun & diffuse sky UVR measurements
PiSpec Spectrometer	UVR absorption by SO_2_ in volcanic plume
**Mauna Loa Observatory Instruments**	
UVB-1 UVB Broadband Radiometer	UVI and cosine comparison for PMA1102
7-channel UV Shadowband Radiometer	UVR at 300, 305, 311, 317, 325, 332 & 368 nm
Brewer Spectrophotometers (009 & 119)	UVI comparison for PMA1102 and total ozone

**Table 3:** Maximum and minimum mannequin head sensor UVI values plotted in Figure 5.

Mannequin Sensor	Morning Peak UVI	Noon Peak UVI	Noon Minimum UVI
**Left Cheek**	5.5	1.6	1.1
**Left Ear**	5.0	1.1	0.6
**Left Eye**	6.5	1.6	1.1

**Table 4:** Maximum and minimum mannequin head sensor UVI values plotted in Figure 6a,c.

Site	Global UVI	Morning Peak UVI	Noon Peak UVI	Noon Minimum UVI
**Kona**	12.8	5.2	2.8	2.7
**MLO**	14.2	5.5	1.6	1.1

**Table 5:** Comparison of direct and diffuse UVI measurements near solar noon at the Old Kona Airport Park beach (22 July 2018) and MLO (12 August 2018). The Kona sky was overlain with hazy vog with distant cumulus clouds very low over the west horizon; the MLO sky was clean and clear.

Site	Noon SZA	Global UVI	Direct	Diffuse	Cheek UVI	Direct	Diffuse	Surface Reflectance UVR
Kona	3.6°	12.6	38%	62%	2.8	--	~100%	Sand (5.9%)
MLO	4.6°	16	69%	31%	2.4	--	~100%	Lava (20%)

**Table 6:** Cameras used in the UVI survey and their principle purposes.

Camera	Purpose
Olympus Tough water proof	Apparatus and general imagery
Canon G9 with sun occluder	Solar aureole imagery
Sony α6000 with 190° lens	Full sky imagery
Mi Sphere VR 360°	Full sphere imagery of the sky and surface
Custom UV camera	Color-coded images of UVR on the mannequin head

## References

[1] USDA UV-B Monitoring and Research Program, Colorado State University 2018. https://uvb.nrel.colostate.edu/UVB/index.jsf.

[2] Nullet D., Juvik J.O. (1997). Measured Altitudinal Profiles of UV-B Irradiance in Hawai’i. Phys. Geogr..

[3] Grainger R.G., Basher R.E., McKenzie R.L. (1993). UV-B Robertson-Berger characterization and field calibration. App. Opt..

[4] Voiland A. (2018). Tracking the Kilauea Eruption. NASA Earth Observatory.

[5] Kerr J.B. (2005). Understanding the factors that affect surface ultraviolet radiation. Opt. Eng..

[6] Mims F.M., Frederick J.E. (1994). Cumulus Clouds and UV-B. Nature.

[7] Moan J., Dahlback A., Setlow R.B. (1999). Epidemiological support for an hypothesis for melanoma induction indicating a role for UVA radiation. Photochem. Photobiol..

[8] Grant W.B. (2016). The UVB-vitamin D3-skin pigmentation hypothesis is alive and well. Am. J. Phys. Anthropol..

[9] Moukayed M., Grant W.B. (2013). Molecular link between vitamin D and cancer prevention. Nutrients.

[10] Grant W.B. (2013). A multicountry ecological study of cancer incidence rates in 2008 with respect to various risk-modifying factors. Nutrients.

[11] (2018). World Health Organization, Ultraviolet Radiation. https://www.who.int/uv/sun_protection/en/.

[12] Kerr J.B., McElroy C.T., Tarasick D.W., Wardle D.I., Hudson R.D. (1994). The Canadian Ozone Watch and UV-B Advisory programs. Ozone in the troposphere and stratosphere, Part 2. Proceedings of the Quadrennial Ozone Symposium.

[13] Fioletov V., Kerr J., Fergusson A. (2010). The UV Index: Definition, Distribution and Factors Affecting It. Can. J. Public Health.

[14] World Health Organization (2002). Global Solar Uv Index: A Practical Guide.

[15] World Meteorological Organization (2018). Global Atmospheric Watch (GAW): UV Radiation.

[16] McKinlay A.F., Diffey B.L. (1987). A Reference Action Spectrum for Ultraviolet Induced Erythema in Human Skin. CIE J..

[17] Webb A.R., Slaper H., Koepke P., Schmalvieser A. (2011). Know your standard: clarifying the CIE erythema spectrum. Photochem. Photobiol..

[18] He H., Fioletov V.E., Tarasick D.W., Mathews T.W., Long C. (2013). Validation of Environment Canada and NOAA UV Index Forecasts with Brewer Measurements from Canada. J. Appl. Meteorol. Climatol..

[19] Cadet J.M., Bencherif H., Portafaix T., Lamy K., Ncongwane K., Coetzee G.J.R., Wright C.Y. (2017). Comparison of Ground-Based and Satellite-Derived Solar UV Index Levels at Six South African Sites. Int. J. Environ. Res. Public Health.

[20] Reichrath J. (2009). Skin cancer prevention and UV-protection: how to avoid vitamin D-deficiency?. Br. J. Dermatol..

[21] McKenzie R.L., Lucas R.M. (2018). Reassessing Impacts of Extended Daily Exposure to Low Level Solar UV Radiation. Nat. Sci. Rep..

[22] Gies P., van Deventer Green A.C., Sinclair C., Tinker R. (2018). Review of the Global Solar UV Index 2015 Workshop Report. Health Phys..

[23] Davis A., Deane G.H.W., Diffey B.L. (1976). Possible dosimeter for ultraviolet radiation. Nature.

[24] Downs N.J., Kimlin M.G., Parisi A.V., McGrath J.J. (2001). Modelling human facial UV exposure. Rad. Prot. Australas..

[25] Parisi A.V., Eley R., Downs N. (2012). Determination of the Usage of Shade Structures via a Dosimetry Technique. Photochem. Photobiol..

[26] Downs N., Parisi A. (2007). Three dimensional visualisation of human facial exposure to solar ultraviolet. Photochem. Photobiol. Sci..

[27] Wang F., Yu J.M., Yang D.Q., Gao Q., Hua H., Liu Y. (2017). Distribution of Facial Exposure to Non-melanoma Biologically Effective UV Irradiance Changes by Rotation Angles. Biomed. Environ. Sci..

[28] Mims F.M. (2018). Sunburn Sensors. MAKE Mag..

[29] Bigelow D.S., Slusser J.R., Beaubien A.F., Gibson J.H. (1998). The USDA Ultraviolet Radiation Monitoring Program. Bull. Am. Meteorol. Soc..

[30] McKenzie R.L., Matthews W.A., Johnston P.V. (1991). The relationship between erythemal UV and ozone, derived from spectral irradiance measurements. Geophys. Res. Lett..

[31] Seckmeyer G., Bais A., Bernhard G., Blumthaler M., Booth C.R., Lantz K., McKenzie R.L. (2008). Instruments to Measure Solar Ultraviolet Radiation, Part 2: Broadband Instruments Measuring Erythemally Weighted Solar Irradiance.

[32] Krins A., Bolsée B., Dörschel B., Gillotay D., Knuschke P. (2000). Angular Dependence of the Efficiency of the UV Sensor Polysulphone Film. Radiat. Prot. Dosim..

[33] Mims F.M. (1994). Dual-Channel Solar Radiometer with Interchangeable UV and Visible Sensors.

[34] Swift N., Hülsen G., Nield K., Gröbner J., Hamlin J. (2013). Calibration of erythemally weighted broadband instruments: A comparison between PMID/WRC and MSL. AIP Conference Proceedings.

[35] Hülsen G., Gröbner J. (2007). Characterization and calibration of ultraviolet broadband radiometers measuring erythemally weighted irradiance. App. Opt..

[36] Tamar E., Sutton A.J. Volcanic Air Pollution Hazards in Hawai’i. USGS Fact Sheet 2017–3017.

[37] Hawai’i Volcano Observatory (2018). Kīlauea 2018 events mark a watershed for volcano science. Volcano Watch.

[38] Wilkes T.C., McGonigle A.J.S., Willmott J.R., Pering T.D., Cook J.M. (2017). Low-cost 3D printed 1 nm resolution smartphone sensor-based spectrometer: Instrument design and application in ultraviolet spectroscopy. Opt. Lett..

[39] Zerefos C.S., Mantis H.T., Bais A.F., Ziomas I.C., Zoumakis N. (1986). Solar ultraviolet absorption by sulphur dioxide in Thessaloniki, Greece. Atmosphere-Ocean.

[40] Komhyr W.D., Evans R.D. (1980). Dobson spectrophotometer measurement errors caused by interfering absorbing species such as SO2, NO2 and photochemically produced O3, in polluted air. Geophys. Res. Lett..

[41] Morys M., Mims F.M., Hagerup S., Anderson S.E., Baker A., Kia J., Walkup T. (2001). Design, calibration, and performance of MICROTOPS II handheld ozone monitor and Sun photometer. J. Geophys. Res..

[42] U.S. Geological Survey Hawaiian Volcano Observatory Scientists and Affiliates (2018). Volcano Watch: Significant Changes in Air Quality. Big Island Now.

[43] (2018). National Park Service, Hawai’i Volcanoes National Park News Release, Keauhou Ranch Fire. https://www.nps.gov/havo/keauhou-ranch-fire.htm.

[44] Waters L. (2018). Personal communication.

[45] Mims F.M. (1990). How to Monitor Ultraviolet Radiation from the Sun. Sci. Am..

[46] Mims F.M. (1996). Significant Reduction in UV-B Caused by Smoke from Biomass Burning in Brazil. Photochem. Photobiol..

[47] Mims F.M. (2005). Avian Influenza and UV-B Blocked by Biomass Smoke. Environ. Health Perspect..

[48] Mims F.M. (2009). How to photograph the solar aureole. Make Mag..

[49] Wilkes T.C., McGonigle A.J., Pering T.D., Taggart A.J., White B.S., Bryant R.G., Willmott J.R. (2016). Ultraviolet imaging with low cost smartphone sensors: Development and application of a raspberry Pi-based UV camera. Sensors.

[50] Mori T., Burton M. (2006). The SO_2_ camera: A simple, fast and cheap method for ground-based imaging of SO_2_ in volcanic plumes. Geophys. Res. Lett..

[51] Kern C., Werner C., Elias T., Sutton A.J., Lübcke P. (2013). Applying UV cameras for SO_2_ detection to distant or optically thick volcanic plumes. J. Volcanol. Geotherm. Res..

[52] Kern C., Lübcke P., Bobrowski N., Campion R., Mori T., Smekens J.-F., Stebel K., Tamburello G., Burton M.R., Platt U. (2015). Intercomparison of SO_2_ camera systems for imaging volcanic gas plumes. J. Volcanol. Geotherm. Res..

[53] Blumthaler M. (2018). UV Monitoring for Public Health. Int. J. Environ. Res. Public Health.

[54] McKenzie R., Blumthaler M., Diaz S., Fioletov V., Herman J., Seckmeyer G., Smedley A., Webb A. (2014). Rationalizing Nomenclature for UV Doses and Effects on Humans, CIE and WMO.

